# Pre-Diagnostic Leukocyte Genomic DNA Methylation and the Risk of Colorectal Cancer in Women

**DOI:** 10.1371/journal.pone.0059455

**Published:** 2013-04-01

**Authors:** Hongmei Nan, Edward L. Giovannucci, Kana Wu, Jacob Selhub, Ligi Paul, Bernard Rosner, Charles S. Fuchs, Eunyoung Cho

**Affiliations:** 1 Division of Cancer Epidemiology, Department of Epidemiology and Public Health, University of Maryland School of Medicine, Baltimore, Maryland, United States of America; 2 Channing Division of Network Medicine, Department of Medicine, Brigham and Women’s Hospital and Harvard Medical School, Boston, Massachusetts, United States of America; 3 Department of Medical Oncology, Dana-Farber Cancer Institute and Harvard Medical School, Boston, Massachusetts, United States of America; 4 Department of Nutrition, Harvard School of Public Health, Boston, Massachusetts, United States of America; 5 Department of Epidemiology, Harvard School of Public Health, Boston, Massachusetts, United States of America; 6 Jean Mayer USDA Human Nutrition Research Center on Aging at Tufts University, Boston, Massachusetts, United States of America; 7 Department of Biostatistics, Harvard School of Public Health, Boston, Massachusetts, United States of America; CEA - Institut de Genomique, France

## Abstract

**Background:**

Abnormal one-carbon metabolism may lead to general genomic (global) hypomethylation, which may predispose an individual to the development of colorectal neoplasia.

**Methods:**

We evaluated the association between pre-diagnostic leukocyte genomic DNA methylation level and the risk of colorectal cancer in a nested case-control study of 358 colorectal cancer cases and 661 matched controls within the all-female cohort of the Nurses’ Health Study (NHS). Among control subjects, we further examined major plasma components in the one-carbon metabolism pathway in relation to genomic DNA methylation level. Liquid chromatography/tandem mass spectrometry was used to examine leukocyte genomic DNA methylation level. We calculated odds ratios (ORs) and 95% confidence intervals (95% CIs) using logistic regression.

**Results:**

Overall genomic DNA methylation level was not associated with the risk of colorectal cancer (*p* for trend, 0.45). Compared with women in the lowest quintile of methylation, the multivariate OR of colorectal cancer risk was 1.32 (95% CI, 0.82–2.13) for those in the highest quintile. We did not find significant associations between major plasma components of one-carbon metabolism or risk factors for colorectal cancer and genomic DNA methylation level (all *p* for trend >0.05). Also, neither one-carbon metabolism-related plasma components nor well-known risk factors for colorectal cancer modified the association between genomic DNA methylation level and the risk of colorectal cancer (all *p* for interaction >0.05).

**Conclusions:**

We found no evidence that hypomethylation of leukocyte genomic DNA increases risk of colorectal cancer among women. Additional studies are needed to investigate the association between pre-diagnostic genomic DNA methylation level and colorectal cancer risk among diverse populations.

## Introduction

One-carbon metabolism, or methyl-group metabolism, is a critical pathway in cancer epigenetics [1]. It comprises a network of interrelated biochemical reactions involved in the transfer of methyl groups from one compound to another, which among other effects can also affect the level of DNA methylation. Abnormal one-carbon metabolism may result in general genomic (global) hypomethylation, which may predispose an individual to the development of neoplasia [2]. Few epidemiologic studies have examined pre-diagnostic genomic DNA methylation status in white blood cells in relation to cancer risk. A cross-sectional study found that higher genomic methylation of leukocyte DNA is associated with a reduced risk of colorectal adenoma, a precursor for colorectal cancers [3].

We evaluated the influence of leukocyte genomic DNA methylation status and its interaction with one-carbon metabolism-related nutrients, genetic polymorphisms, and other factors on the risk of colorectal cancer in a nested case-control study within the Nurses’ Health Study (NHS).

## Materials and Methods

### Study Population

The NHS enrolled 121,700 female registered nurses aged 30–55 years in 1976; for more detail, please refer to [4]. We have sent follow-up questionnaires to the cohort biennially to update information regarding lifestyle factors and to ascertain new diagnoses of major illnesses. The follow-up rate for this cohort remains above 90% [5]. In 1989–90, we collected blood samples from 32,826 participants in this cohort. The samples were collected in tubes with heparin and sent to us by overnight courier in chilled containers. On receipt, the bloods were centrifuged, aliquoted, and stored at −70°C.

The procedures and protocols of the study were approved by the Institutional Review Boards at Brigham and Women’ Hospital.

### Selection of Colorectal Cancer Cases and Controls

We requested permission from cohort members who reported colorectal cancer on our biennial questionnaires to obtain medical records and pathology reports. We identified fatal cases from the National Death Index and from next-of-kin [6]. Study physicians blinded to exposure data reviewed all medical records to confirm cases of colorectal cancer. We included incident colorectal cancer cases who had provided a blood sample prior to cancer diagnosis. We excluded the small number of cancers that were not adenocarcinomas as well as carcinomas in situ. We randomly selected one or two controls within the same cohort from participants who had also provided blood samples but were free of colorectal cancer at the time when the case was diagnosed. Controls were matched to each case by age (within 3 years of birth), month/year of blood sampling (95% of cases and controls were matched within one month of blood sampling), and fasting status (<8 versus ≥8 hours since last meal). In total, 358 incident colorectal cancer cases diagnosed after the 1990 blood collection until October 2008 and 661 matched controls were included in our analysis.

### Documentation of Life-style and Genetic Data

For the cases and controls, dietary data have been collected. Briefly, a semiquantitative food-frequency questionnaire (FFQ) with approximately 60 items was sent to the entire NHS cohort in 1980. An expanded FFQ with approximately 130 food items was administered to women in 1984, 1986, and every 4 years thereafter. Participants were asked how often on average they had consumed each type of food or beverage, including alcoholic beverages (beer, wine, and liquor) during the past year. Serving sizes were specified for each food in the FFQ. The questionnaire had nine possible responses, ranging from never or less than once per month to six or more times per day. Responses on frequencies of a specified serving size for each food item were converted to average daily intakes. Quantities of nutrients from foods were calculated by multiplying the reported frequency of each food by the nutrient content of one serving of that food primarily on the basis of the US Department of Agriculture Nutrient Database [7]. For each nutrient, energy intake was adjusted for using the nutrient residual method [8].

Similarly, nondietary data have also been collected prospectively for both cases and controls. Briefly, information on weight, physical activity, aspirin use, history of endoscopy, and postmenopausal hormone use was updated roughly every 2 years. Information on family history of colorectal cancer in parents or siblings was obtained in the 1982 questionnaire and was updated in 1988, 1992, 1996, and 2000. The average number of cigarettes smoked per day when subjects started to smoke, the average number of cigarettes smoked per day in the first 5 years of smoking, the age when subjects last smoked, and height were assessed at baseline.

For a subset of the cases and controls in this study, we have also assessed the plasma levels of major components in the one-carbon metabolism pathway, including folate, vitamin B_6_, vitamin B_12_, and homocysteine [9]. In brief, plasma concentrations of folate and vitamin B_12_ were determined using a radioassay kit (Bio-Rad, Richmond, CA), which uses a dual-radioisotope competitive protein-binding assay. The kit simultaneously measures plasma folate and vitamin B_12_. We used plasma concentrations of pyridoxal 5′-phosphate (PLP) to determine vitamin B_6_ levels, because PLP is the main active form of vitamin B_6_. PLP concentrations were determined using an enzymatic procedure based on radioactive tyrosine and the apoenzyme tyrosine decarboxylase, as described by Shin et al. [10]. Total plasma homocysteine and cysteine concentrations are measured using high-performance liquid chromatography with fluorescence detection as described by Araki and Sako [11]. The mean coefficients of variation for 75 pairs of replicate plasma samples in the NHS were 6.5% for folate, 7.2% for vitamin B_6_, 7.3% for vitamin B_12_, and 7.9% for homocysteine [12].

Methylene-tetrahydrofolate reductase (MTHFR) is a critical enzyme in the one-carbon metabolism. Two nonsynonymous single nucleotide polymorphisms (SNPs) in the *MTHFR* gene (C677T [rs1801133] and A1298C [rs1801131]) have previously been reported to alter MTHFR activity, and are also associated with risk of colorectal neoplasia [13], [14], [15]. For these two SNPs in the *MTHFR* gene, we have previously ascertained their genotypes in a subset of the cases and controls in this study [16]. Briefly, these two SNPs were genotyped by the 5 nuclease assay (TaqMan®), using the ABI PRISM 7900HT Sequence Detection System (Applied Biosystems, Foster City, CA). TaqMan® primers and probes were designed using the Primer Express® Oligo Design software v2.0 (ABI PRISM). Laboratory personnel were blinded to case-control status, and 10% blinded quality-control samples (duplicated samples) were inserted to validate genotyping procedures; concordance for the blinded quality-control samples was 100%. Primers, probes, and conditions for the genotyping assay are available upon request. We confirmed that these two SNPs were in Hardy-Weinberg equilibrium among the controls.

### Genomic DNA Methylation Assay

Laboratory personnel were blinded to case-control status, and the matched cases and controls were handled identically and together, shipped in the same batch, and assayed in the same analytical run. In addition, the order within each case-control pair was random.

A liquid chromatography/tandem mass spectrometry (LC/MS/MS) method was used to determine leukocyte genomic DNA methylation status [17] using the ABI 3200 QTRAP LC/MS/MS from Applied Biosystems. Briefly, 100 nanograms of DNA was enzymatically hydrolyzed by sequential digestion with three nucleases. DNA hydrolysates were then injected onto an analytic column and subsequently separated by reversed-phase high-performance liquid chromatography in isocratic mode. The four major DNA bases and 5-methyl-2′-deoxycytidine were resolved and eluted in a short time run. 5-methyl-2′-deoxycytidine was identified by spectral analysis of chromatographic peaks by isotope-labeled compounds as internal standard. The absolute amount (in nanograms) of 5-methyl-2′-deoxycytidine (mCyt) per 100 nanograms of DNA was determined. Percent genomic DNA methylation status was defined as the percent of mCyt in total Cyt and was used for the statistical analyses. For the percent genomic DNA methylation level, the coefficient of variance (CV) was 4% and intraclass correlation coefficient (ICC) was 0.34.

### Statistical Analyses

We categorized the participants into quintiles based on percent genomic DNA methylation level, using the first (lowest) quintile as the reference group. We used conditional logistic regression to estimate odds ratios (ORs) and corresponding 95% confidence intervals (95% CIs) for the association between genomic methylation status and total colorectal cancer risk. Multivariate ORs were obtained from the conditional logistic regression models, adjusting for known or suspected colorectal cancer risk factors. Tests for trend were conducted by assigning the median value of each quintile of methylation status among controls to both cases and controls in each category and modeling it as a continuous variable.

To determine whether the association between genomic methylation status and colorectal cancer risk differed by levels of plasma folate or homocysteine, total folate intake or alcohol consumption, smoking status, family history of colorectal cancer, age at blood draw, year of diagnosis, as well as two SNPs in the *MTHFR* gene (C677T [rs1801133] and A1298C [rs1801131]), we conducted analyses stratified by these variables. The statistical significance of interaction was assessed by adding cross-product terms of these variables and methylation status into the logistic regression model, adjusting for matching factors as well as risk factors for colorectal cancer.

Among control subjects, a linear regression model was employed to examine the association between genomic methylation status and major factors involved in one-carbon metabolism and known and suspected risk factors for colorectal cancer (plasma folate, vitamin B_6_, vitamin B_12_, homocysteine, total folate or alcohol intake, smoking status, family history of colorectal cancer, age at blood draw, as well the two SNPs in the *MTHFR* gene). Linear regression models were adjusted for matching factors as well as risk factors for colorectal cancer. To attenuate the influence of extreme values of the components, we calculated the geometric mean of the methylation levels and corresponding 95% CIs for each level of variables and modeled it as a continuous variable. For each one-carbon component, tests for trend were conducted by modeling the median value of each quintile as a continuous variable. All statistical analyses were two-sided and carried out using SAS V9.2 (SAS Institute, Cary, NC). A p-value<0.05 was considered statistical significant.

## Results

The baseline characteristics of the 358 colorectal cancer cases and 661 controls are presented in Table 1. The mean age at diagnosis of colorectal cancer cases was 68.3 years. Compared with controls, colorectal cancer cases were more likely to have family history of colorectal cancer, more likely to smoke, consumed higher amounts of alcohol, and had lower intakes of calcium, vitamin D, and folate, and were less physically active. Among controls, the level of these colorectal cancer-related factors was not significantly altered according to quintiles of genomic DNA methylation level (Table 2).

**Table 1:** Characteristics of colorectal cancer cases and controls in the nested case-control study within the Nurses’ Health Study.

Characteristic	Cases (n = 358)	Controls (n = 661)
Age at diagnosis (years, mean)	68.3	NA
Age at blood draw (years, mean)	59.0	59.0
Regular aspirin use (%)	46.4	46.4
Body mass index (kg/m^2^, mean)^a^	25.9	25.4
Physical activity (METs -hours/wk, mean)^b^	16.0	17.0
Colorectal cancer in a parent or sibling (%)	15.6	14.1
Former or current smoker (%)	58.1	56.7
Alcohol consumption (g/day, mean)	5.9	5.2
Former or current postmenopausal hormone use (%)	50.8	50.5
Beef, pork, or lamb as a main dish (servings/wk, mean)	1.1	1.1
Total calcium intake (mg/, mean)^c^	747.9	757.2
Total vitamin D intake (IU/d, mean)^c^	216.8	227.1
Total folate intake (µg/d, mean)^c^	424.5	456.2

a Body mass index is weight in kilograms divided by the square of the height in meters.

b MET denotes metabolic equivalent. Met –hours = sum of the average time/wk in each activity x MET value of each activity. One MET, the energy spent sitting quietly, is equal to 3.5 ml of oxygen uptake per kilograms of body weight per minute for a 70-kg adult.

c Nutrient values (calcium, vitamin D, and folate) represent the mean of energy-adjusted intakes.

**Table 2:** Characteristics of colorectal cancer controls by levels of leukocyte genomic DNA methylation in a case-control study nested within the Nurses’ Health Study.

	Methylation (quintiles; range, % )				
Characteristic	1	2	3	4	5
	(3.628–4.107)	(4.108–4.196)	(4.198–4.272)	(4.273–4.353)	(4.354–4.788)
Number of controls	131	134	131	133	132
Age at blood draw (years, mean)	58.8	59.6	58.8	59.3	58.3
Regular aspirin use (%)^a^	41.0	42.8	53.7	44.9	47.6
Body mass index (kg/m^2^, mean)^b^	25.7	25.1	25.6	25.7	25.2
Physical activity (METs -hours/wk, mean)^c^	14.8	16.8	18.5	17.8	16.6
Colorectal cancer in a parent or sibling (%)	9.6	17.6	14.6	16.3	13.0
Former or current smoker (%)	52.6	63.3	53.1	58.8	56.4
Alcohol consumption (g/d, mean)	4.4	6.0	5.4	5.1	5.2
Former or current postmenopausal hormone use (%)	45.1	53.7	51.0	51.2	51.4
Beef, pork, or lamb as a main dish (servings/wk, mean)	1.1	1.0	1.0	1.1	1.0
Total calcium intake (mg/d, mean)^d^	761.0	736.4	749.4	783.2	774.0
Total vitamin D intake (IU/d, mean)^d^	217.1	221.5	236.3	239.8	223.1
Total folate intake (ug/d, mean)^d^	449.5	467.9	442.8	450.0	469.1

a Regular aspirin use was defined as intake of at least two 325-mg tablets per week in the NHS.

b The body mass index is weight in kilograms divided by the square of the height in meters.

c MET denotes metabolic equivalent. Met -hours = sum of the average time/wk in each activity x MET value of each activity. One MET, the energy spent sitting quietly, is equal to 3.5 ml of oxygen uptake per kilograms of body weight per minute for a 70-kg adult.

d Nutrient values (calcium, folate, and vitamin D) represent the mean of energy-adjusted intakes.

Overall the percent genomic DNA methylation levels were not associated with risk of colorectal cancer (Table 3). Compared with women in the lowest quintile of methylation, the multivariate OR of colorectal cancer risk was 1.32 (0.82–2.13) for those in the highest quintile (*p* for trend, 0.45). Similarly, we did not find significant associations between methylation status and risk of colorectal cancer by subsites (i.e., colon, rectum, proximal colon, and distal colon) (Table S1).

**Table 3:** Association between leukocyte genomic DNA methylation level and colorectal cancer risk in the nested case-control study.

Methylation (%)	1^st^ quintile(3.628–4.107)	2^nd^ quintile(4.108–4.196)	3^rd^ quintile(4.198–4.272)	4^th^ quintile(4.273–4.353)	5^th^ quintile(4.354–4.788)	*P* for trend
No. of cases/controls	64/131	82/134	69/131	66/133	77/132	
Median (%)	4.03	4.16	4.24	4.31	4.43	
Crude OR (95% CI)	1.00	1.37 (0.89–2.09)	1.18 (0.75–1.83)	1.07 (0.68–1.68)	1.29 (0.82–2.03)	0.50
Multivariate OR (95% CI)	1.00	1.32 (0.84–2.08)	1.25 (0.78–2.01)	1.09 (0.68–1.75)	1.32 (0.82–2.13)	0.45

Multivariate ORs are adjusted for race, height (continuous), pack-years of smoking (continuous), body mass index (continuous), physical activity (in quartiles), family history of colorectal cancer (yes or no), history of colonoscopy or sigmoidoscopy (yes or no), alcohol intake (continuous), intake of red and processed meat (in quartiles), vitamin D intake (continuous), calcium intake (continuous), and aspirin use (non-users vs. ever users).

The association between methylation status and colorectal cancer risk did not vary by either one-carbon metabolism-related factors or well-known risk factors for colorectal cancer (all *p* for interactions >0.05) (Table S2).

We further examined the association of major components of the one-carbon metabolism and colorectal cancer-related factors with genomic DNA methylation status (Table S3). None of these variables was associated with methylation status (all *p* for trends >0.05).

In colorectal tumor tissue from a subset of the colorectal cancer cases [18], [19], we assessed levels of long interspersed nucleotide element-1 (LINE-1) methylation, which may be correlated with global DNA methylation levels. We evaluated the correlation between leukocyte genomic DNA methylation levels and tumor LINE-1 methylation levels among the cases with LINE-1 values; the Pearson correlation coefficient was 0.11 (*p* = 0.19) in 141 samples.

## Discussion

DNA methylation plays critical mechanistic roles in gene regulation and cellular differentiation. Preserving the patterns of DNA methylation is a major factor in the epigenetic control of genomic stability and the regulation of gene transcription [20], [21]. In eukaryotic cells, DNA methylation occurs at the carbon-5 position of cytosine, i.e., a methyl group is transferred from the methyl donor S-adenosylmethionine to the cytosine-5 carbon by DNA methyltransferases. Those methyltranferases predominantly recognize the sequence of cytosine-guanine (CpG), and over 70% of the cytosine residues in CpG dinucleotides of mammalian genomic DNA are methylated [22].

An altered genomic DNA methylation profile is frequently found in cancers. Previous studies have shown that abnormal DNA methylation is common in neoplastic cells, including widespread genomic hypomethylation and hypermethylation of CpG clusters known as CpG islands, mostly residing within gene promoter regions [22]. Such changes in DNA methylation can contribute to carcinogenesis by promoting the expression of oncogenes and transcriptional silencing of tumor suppressor genes and/or making critical tumor suppressor genes more susceptible to DNA damage [2], [23], [24].

Genomic DNA hypomethylation is an early and consistent event in colorectal carcinogenesis [25], [26]. For example, a case-control study of 35 cases of colorectal adenoma (a premalignant condition of colorectal cancer) found that both colonic and leukocyte DNA hypomethylation were significantly associated with an increased risk of colorectal adenoma [27]. Similarly, using the same LC/MS method used here for methylation assay, a cross-sectional study conducted by Lim et al. found that leukocyte genomic DNA hypomethylation was associated with an increased risk of colorectal adenoma [3]. Compared with those in the highest tertile of genomic methylation, participants in the lowest tertile had an OR of 5.88 (95% CI, 2.04–16.67) for the risk of colorectal adenoma. In contrast, for colorectal cancer, a case-control study of 28 cases and 76 controls did not find an association with either colonic or leukocyte genomic DNA methylation level [27]. We also found no association between pre-diagnostic genomic DNA methylation status and colorectal cancer risk. Similarly, using essentially the same LC/MS method, Huang et al. found no clear association between leukocyte methylation level and colorectal cancer risk [28]. We note, however, that the evidence regarding genomic DNA hypomethylation and cancer risk is controversial, because regional hypermethylation may also be related to increased cancer risk [22], [29], [30], [31].

One issue to discuss is why our results differ from those of Lim et al. [3]. We utilized the same assay, and although the absolute methylation levels were slightly lower in our study, the ranges were very similar. Thus, the ranking of the subjects should be the same. Interestingly, Lim et al. [3] detected the association with methylation level only in non-advanced adenomas, though they had limited numbers of advanced adenomas. It is therefore plausible that genomic hypomethylation may be more relevant for a sub-group of adenomas less likely to progress to malignancy.

General genomic DNA hypomethylation could to some extent be derived from abnormal one-carbon metabolism [2]. One of the central elements in the methyl-group metabolism pathway is folate, which donates a single carbon to homocysteine to form methionine, which is then converted to S-adenosylmethionine, to which DNA transfers the methyl group [32], [33], [34]. To date, folate and other major factors involved in one-carbon metabolism including vitamin B_6,_ vitamin B_12,_ homocysteine, and MTHFR (a major folate metabolic enzyme) have been associated with several cancer sites including colon, breast, pancreas, cervix, bronchus, and leukaemia [35]; the evidence of an association with colorectal cancer is most compelling in both animal and human studies [36].

Abnormal levels of major components in the one-carbon metabolism may alter the status of genomic DNA methylation and further influence colorectal carcinogenesis [2], [36]. For instance, a previous study of 33 elderly women found that moderate folate depletion reduces leukocyte genomic DNA methylation level [37]. In addition, two SNPs in the *MTHFR* gene (C677T and A1298C) have been associated with genomic DNA methylation levels [13], [38], [39]. Carriers of the 677TT genotype had lower leukocyte genomic DNA methylation level than those with the 677CC wild-type genotype, and genomic DNA methylation level was also associated with an increased risk of colorectal adenoma and cancer, especially in subjects with lower folate levels [14], [38], [39]. Moreover, a recent study by Huang et al. [28] reported an inverse association between natural folate intake and colorectal cancer among individuals with the highest methylation level (*p* for interaction, 0.003). In our study, major one-carbon metabolism components including folate were not found to affect leukocyte genomic DNA methylation status or the association between genomic DNA methylation level and colorectal cancer risk. However, it has to be noted that some of the markers in the one-carbon metabolism are involved not only in DNA methylation but also in multiple other pathways [40].

The LC/MS method used in this study to assess global DNA methylation level in blood leukocytes differs from the assessment of LINE-1 methylation status in a subsample of colorectal tumor tissues [19]. LINE-1 CpG island methylation status was measured using bisulfite-treated DNA, utilizing PCR and subsequent pyrosequencing. Methylation levels of LINE-1 was calculated by the amounts of “C” relative to the sum of the amounts of “C” and “T” at each CpG site. We note that the methylation levels in blood versus tissue specimens have different implications. The methylation status in blood may represent the body’s systemic methylation status rather than the status in a specific organ. We did not find a significant relationship between global DNA methylation level in blood and LINE-1 methylation level in colorectal tumor tissue. A recent study of bladder cancer [41] found that the mean percent LINE-1 methylation level measured in buffy coat was correlated with that in serum, but not with that in tumor tissue, suggesting that methylation level measured in blood may not reflect tissue level.

We acknowledge that there are various methods for assessing genomic DNA methylation status in blood. However, the current study as well as the other two studies [3], [28] conducted to date on the association between genomic DNA methylation level in blood leukocyte and the risk of colorectal adenoma or colorectal cancer have used essentially the same LC/MS method to measure global DNA methylation level. Furthermore, using the same method, neither our study nor the study conducted by Huang et al. [28] found a significant association between pre-diagnostic genomic DNA methylation level and colorectal cancer risk, while the study conducted by Lim et al. [3] identified an association between higher genomic methylation of leukocyte DNA and reduced risk of colorectal adenoma.

Our study has several strengths. First, we measured pre-diagnostic leukocyte genomic DNA methylation level among individuals from a large prospective cohort, ensuring that the measure of DNA methylation level preceded development of colorectal cancer by up to 32 years. Second, our case-control study was nested within a large well-characterized cohort in which matched controls were selected from the same cohort, minimizing the likelihood of population stratification or selection bias [42]. Third, repeated measurements of both dietary and non-dietary factors allowed us to use repeated and updated information on those confounding factors, reducing the likelihood of measurement error.

A potential limitation of this study is the lack of generalizability of the study population, because our participants are an older, non-random sample of US women. However, it is unlikely that the biological relation between DNA methylation level and colorectal cancer risk in our cohort will differ appreciably from US women in general. In previous analyses from the NHS, we detected associations for colorectal cancer and other illnesses that are very similar to those found in other broadly based US populations. Though residual confounding is a concern in observational studies, adjustment for multivariate colorectal cancer risk factors only minimally influenced our findings, suggesting little potential for residual or uncontrolled confounding. The statistical power for the sub-groups analyses was modest; additional studies are warranted to confirm the associations observed in the present study.

In conclusion, our study does not support the hypothesis that pre-diagnostic hypomethylation of leukocyte genomic DNA increases risk of colorectal cancer. Additional studies are needed to investigate the association between pre-diagnostic genomic DNA methylation level and colorectal cancer risk among diverse populations.

## Supporting Information

Table S1 **Association between genomic DNA methylation level and the risk of colorectal cancer according to subsites of large bowel.**
(DOCX)

Table S2 **Risk for colorectal cancer according to genomic DNA methylation status, stratified by one-carbon metabolism-related factors.**
(DOCX)

Table S3 **Association of one-carbon metabolism and colorectal cancer-related factors with percent genomic DNA methylation levels among controls.**
(DOCX)
