# Supplementary material for: Pre-Diagnostic Leukocyte Genomic DNA Methylation and the Risk of Colorectal Cancer in Women
Source: PLoS One. 2013 Apr 1;8(4):e59455. doi: 10.1371/journal.pone.0059455 (PMC3613344; doi:10.1371/journal.pone.0059455)
Supplement: Table S1 — Association between genomic DNA methylation level and the risk of colorectal cancer according to subsites of large bowel. (DOCX) [file pone.0059455.s001.docx]

Supplementary Table 1. Association between genomic DNA methylation level and the risk of colorectal cancer according to subsites of large bowel

| Subsites of cancer (cases/controls) | 1^st^ quintile (3.628-4.107) | 2^nd^ quintile (4.108-4.196) | 3^rd^ quintile (4.198-4.272) | 4^th^ quintile (4.273-4.353) | 5^th^ quintile  (4.354-4.788) | *P* for trend |
| --- | --- | --- | --- | --- | --- | --- |
| Colon (249/620) | 1.00 | 1.15 (0.72-1.85) | 1.02 (0.63-1.66) | 0.96 (0.59-1.56) | 1.17 (0.73-1.89) | 0.74 |
| Rectum (70/620) | 1.00 | 1.52 (0.59-3.95) | 1.94 (0.78-4.82) | 1.37 (0.52-3.58) | 2.09 (0.84-5.20) | 0.16 |
| Proximal (175/620) | 1.00 | 1.00 (0.58-1.72) | 1.01 (0.58-1.76) | 0.91 (0.52-1.59) | 1.26 (0.73-2.16) | 0.51 |
| Distal (94/620) | 1.00 | 1.38 (0.70-2.73) | 1.00 (0.48-2.07) | 0.86 (0.40-1.82) | 0.84 (0.44-1.77) | 0.37 |

Multivariate models are adjusted for age at blood draw, date of blood draw, race, height (continuous), fasting status, pack-years of smoking (continuous), body mass index (continuous), physical activity (in quartiles), family history of colorectal cancer (yes or no), history of colonoscopy or sigmoidoscopy (yes or no), alcohol intake (continuous), intake of red and processed meat (in quartiles), vitamin D intake (continuous), calcium intake (continuous), and aspirin use (non-users vs. ever users).
