# Supplementary material for: Pre-Diagnostic Leukocyte Genomic DNA Methylation and the Risk of Colorectal Cancer in Women
Source: PLoS One. 2013 Apr 1;8(4):e59455. doi: 10.1371/journal.pone.0059455 (PMC3613344; doi:10.1371/journal.pone.0059455)
Supplement: Table S2 — Risk for colorectal cancer according to genomic DNA methylation status, stratified by one-carbon metabolism-related factors. (DOCX) [file pone.0059455.s002.docx]

Supplementary Table 2. Risk for colorectal cancer according to genomic DNA methylation status, stratified by one-carbon metabolism-related factors

| One-carbon metabolism-related factors (No. of cases/controls) | 1^st^ quintile | 2^nd^ quintile | 3^rd^ quintile | 4^th^ quintile | 5^th^ quintile | *P* for trend |
| --- | --- | --- | --- | --- | --- | --- |
|  | (3.628-4.107) | (4.108-4.196) | (4.198-4.272) | (4.273-4.353) | (4.354-4.788) |  |
| Plasma Folate |  |  |  |  |  |  |
| Low (<10.30 ng/ml; 188/351) | 1.00 | 1.01 (0.57-1.79) | 0.71 (0.39-1.29) | 1.13 (0.64-1.99) | 1.24 (0.69-2.21) | 0.47 |
| High (≥10.30 ng/ml; 141/269) | 1.00 | 1.79 (0.87-3.71) | 2.16 (1.03-4.51) | 0.97 (0.44-2.15) | 1.36 (0.65-2.82) | 1.00 |
| *P* for interaction |  |  |  |  |  | 0.62 |
|  |  |  |  |  |  |  |
| Total folate intake |  |  |  |  |  |  |
| Low (≤371.80 µg/d; 189/313) | 1.00 | 1.26 (0.69-2.31) | 1.17 (0.63-2.15) | 1.09 (0.59-2.03) | 1.27 (0.69-2.32) | 0.57 |
| High (>371.80 µg/d; 140/307) | 1.00 | 1.23 (0.63-2.38) | 1.08 (0.54-2.16) | 0.82 (0.41-1.66) | 1.12 (0.57-2.21) | 0.91 |
| *P* for interaction |  |  |  |  |  | 0.80 |
|  |  |  |  |  |  |  |
| Plasma homocystein |  |  |  |  |  |  |
| Low (≤ 9.6 µmol/L; 102/191) | 1.00 | 2.81(0.99-7.96) | 3.41 (1.17-9.96) | 2.21 (0.75-6.52) | 3.26 (1.14-9.32) | 0.10 |
| Middle (>9.6-≤12.6 µmol/L; 119/191) | 1.00 | 0.89 (0.36-2.20) | 0.91 (0.40-2.69) | 1.19 (0.52-2.69) | 1.23 (0.52-2.93) | 0.49 |
| High (>12.6 µmol/L; 119/191) | 1.00 | 1.05 (0.50-2.20) | 0.71 (0.29-1.78) | 0.48 (0.21-1.07) | 0.85 (0.40-1.85) | 0.29 |
| *P* for interaction |  |  |  |  |  | 0.24 |
|  |  |  |  |  |  |  |
| MTHFR C677T |  |  |  |  |  |  |
| CC (84/141) | 1.00 | 1.07 (0.43-2.65) | 1.23 (0.49-3.12) | 1.02 (0.39-2.68) | 1.03 (0.39-2.72) | 0.97 |
| CT or TT (82/171) | 1.00 | 2.18 (0.85-5.63) | 1.80 (0.71-4.60) | 1.61 (0.63-4.16) | 2.49 (0.94-6.60) | 0.13 |
| *P* for interaction |  |  |  |  |  | 0.48 |
|  |  |  |  |  |  |  |
| MTHFR A1298C |  |  |  |  |  |  |
| AA (68/159) | 1.00 | 2.38 (0.84-6.77) | 2.43 (0.84-7.06) | 2.06 (0.71-5.98) | 1.54 (0.45-5.32) | 0.42 |
| AC or CC (105/157) | 1.00 | 0.84 (0.37-1.90) | 0.85 (0.37-1.98) | 1.13 (0.48-2.67) | 1.32 (0.59-2.96) | 0.39 |
| *P* for interaction |  |  |  |  |  | 0.80 |
|  |  |  |  |  |  |  |
| Alcohol intake |  |  |  |  |  |  |
| Non-drinker (64/130) | 1.00 | 2.42 (0.85-6.92) | 1.59 (0.54-4.65) | 1.97 (0.68-5.69) | 1.74 (0.59-5.18) | 0.37 |
| <15 g/d (218/411) | 1.00 | 1.00 (0.58-1.72) | 0.93 (0.53-1.62) | 0.85 (0.48-1.49) | 1.27 (0.74-2.17) | 0.51 |
| ≥15 g/d (47/79) | 1.00 | 1.04 (0.27-3.93) | 1.66 (0.42-6.50) | 0.78 (0.20-3.10) | 0.58 (0.14-2.45) | 0.45 |
| *P* for interaction |  |  |  |  |  | 0.69 |
|  |  |  |  |  |  |  |
| Smoking status |  |  |  |  |  |  |
| Never (137/267) | 1.00 | 1.58 (0.79-3.14) | 1.04 (0.51-2.09) | 0.94 (0.46-1.92) | 1.63 (0.82-3.22) | 0.42 |
| Past (143/270) | 1.00 | 1.23 (0.62-2.43) | 0.98 (0.48-1.98) | 1.04 (0.52-2.08) | 1.05 (0.51-2.14) | 0.92 |
| Current (47/80) | 1.00 | 0.58 (0.14-2.42) | 1.33 (0.30-6.04) | 0.89 (0.20-3.88) | 0.94 (0.22-4.03) | 0.86 |
| *P* for interaction |  |  |  |  |  | 0.56 |
|  |  |  |  |  |  |  |
| Family history of colorectal cancer |  |  |  |  |  |  |
| No (277/530) | 1.00 | 1.30 (0.81-2.09) | 1.09 (0.67-1.77) | 1.12 (0.69-1.82) | 1.27 (0.79-2.04) | 0.49 |
| Yes (52/90) | 1.00 | 0.81 (0.21-3.08) | 1.26 (0.35-4.58) | 0.68 (0.17-2.82) | 1.33 (0.34-5.13) | 0.65 |
| *P* for interaction |  |  |  |  |  | 0.98 |
|  |  |  |  |  |  |  |
| Age at blood draw |  |  |  |  |  |  |
| < 60 yrs (164/310) | 1.00 | 1.26 (0.67-2.39) | 1.17 (0.62-2.21) | 1.03 (0.53-1.98) | 1.13 (0.61-2.11) | 0.91 |
| ≥ 60 yrs (165/310) | 1.00 | 1.28 (0.68-2.41) | 1.40 (0.72-2.70) | 1.14 (0.59-2.21) | 1.67 (0.87-3.21) | 0.19 |
| *P* for interaction |  |  |  |  |  | 0.24 |
|  |  |  |  |  |  |  |
| Year of diagnosis |  |  |  |  |  |  |
| Before 1998 (137/256) | 1.00 | 1.24 (0.61-2.54) | 1.43 (0.70-2.92) | 1.06 (0.50-2.25) | 1.26 (0.59-2.68) | 0.67 |
| 1998 or after (215/394) | 1.00 | 1.22 (0.69-2.16) | 0.87 (0.48-1.59) | 1.03 (0.57-1.87) | 1.20 (0.68-2.10) | 0.69 |
| *P* for interaction |  |  |  |  |  | 1.00 |

Multivariate ORs are adjusted for age at blood draw, date of blood draw, race, height (continuous), fasting status, pack-years of smoking (continuous), body mass index (continuous), physical activity (in quartiles), family history of colorectal cancer (yes or no), history of colonoscopy or sigmoidoscopy (yes or no), alcohol intake (continuous), intake of red and processed meat (in quartiles), vitamin D intake (continuous), calcium intake (continuous), and aspirin use (non-users vs. ever users).
