# Supplementary material for: Pre-Diagnostic Leukocyte Genomic DNA Methylation and the Risk of Colorectal Cancer in Women
Source: PLoS One. 2013 Apr 1;8(4):e59455. doi: 10.1371/journal.pone.0059455 (PMC3613344; doi:10.1371/journal.pone.0059455)
Supplement: Table S3 — Association of one-carbon metabolism and colorectal cancer-related factors with percent genomic DNA methylation levels among controls. (DOCX) [file pone.0059455.s003.docx]

Supplementary Table 3. Association of one-carbon metabolism and colorectal cancer-related factors with percent genomic DNA methylation levels among controls

| One-carbon components (Sample size) | Crude geometric mean of methylation (95% CI*) | *P* for trend | Multivariate geometric mean of methylation (95% CI*) | *P* for trend |
| --- | --- | --- | --- | --- |
| Plasma folate |  |  |  |  |
| 1^st^ quintile (113) | 4.23 (4.20-4.26) | 0.15 | 4.23 (4.20-4.27) | 0.50 |
| 2^nd^ quintile (115) | 4.20 (4.17-4.23) |  | 4.20 (4.17-4.23) |  |
| 3^rd^ quintile (116) | 4.24 (4.21-4.27) |  | 4.24 (4.21-4.27) |  |
| 4^th^ quintile (113) | 4.25 (4.23-4.28) |  | 4.25 (4.22-4.28) |  |
| 5^th^ quintile (115) | 4.23 (4.20-4.26) |  | 4.23 (4.20-4.26) |  |
|  |  |  |  |  |
| Plasma vitamin B6 |  |  |  |  |
| 1^st^ quintile (113) | 4.24 (4.21-4.27) | 0.58 | 4.24 (4.20-4.27) | 0.76 |
| 2^nd^ quintile (114) | 4.23 (4.20-4.26) |  | 4.22 (4.19-4.25) |  |
| 3^rd^ quintile (113) | 4.23 (4.20-4.26) |  | 4.24 (4.21-4.27) |  |
| 4^th^ quintile (114) | 4.23 (4.20-4.26) |  | 4.23 (4.20-4.26) |  |
| 5^th^ quintile (114) | 4.22 (4.19-4.25) |  | 4.22 (4.19-4.25) |  |
|  |  |  |  |  |
| Plasma vitamin B12 |  |  |  |  |
| 1^st^ quintile (114) | 4.22 (4.19-4.25) | 0.61 | 4.22 (4.19-4.25) | 0.36 |
| 2^nd^ quintile (114) | 4.26 (4.23-4.29) |  | 4.26 (4.23-4.29) |  |
| 3^rd^ quintile (115) | 4.23 (4.20-4.26) |  | 4.23 (4.20-4.26) |  |
| 4^th^ quintile (115) | 4.23 (4.20-4.26) |  | 4.21 (4.18-4.25) |  |
| 5^th^ quintile (114) | 4.22 (4.19-4.25) |  | 4.22 (4.19-4.25) |  |
|  |  |  |  |  |
| Plasma homocysteine |  |  |  |  |
| 1^st^ quintile (115) | 4.23 (4.20-4.25) | 0.35 | 4.22 (4.19-4.25) | 0.23 |
| 2^nd^ quintile (114) | 4.22 (4.19-4.25) |  | 4.22 (4.19-4.25) |  |
| 3^rd^ quintile (115) | 4.23 (4.20-4.26) |  | 4.23 (4.20-4.27) |  |
| 4^th^ quintile (114) | 4.22 (4.19-4.25) |  | 4.22 (4.19-4.25) |  |
| 5^th^ quintile (115) | 4.25 (4.22-4.28) |  | 4.25 (4.22-4.29) |  |
|  |  |  |  |  |
| MTHFR C677T |  |  |  |  |
| CC (153) | 4.22 (4.19-4.24) | 0.64 | 4.22 (4.19-4.25) | 0.72 |
| CT (139) | 4.20 (4.18-4.23) |  | 4.20 (4.18-4.23) |  |
| TT (40) | 4.21 (4.16-4.26) |  | 4.22 (4.17-4.27) |  |
|  |  |  |  |  |
| MTHFR A1298C |  |  |  |  |
| AA (170) | 4.20 (4.17-4.22) | 0.05 | 4.20 (4.18-4.23) | 0.12 |
| AC (133) | 4.21 (4.18-4.24) |  | 4.21 (4.18-4.24) |  |
| CC (33) | 4.26 (4.21-4.32) |  | 4.26 (4.21-4.32) |  |
|  |  |  |  |  |
| Total folate intake |  |  |  |  |
| Low (≤371.80 µg/d, 335) | 4.23 (4.21-4.25) | 0.65 | 4.23 (4.21-4.25) | 0.82 |
| High (>371.80 µg/d, 326) | 4.22 (4.21-4.24) |  | 4.22 (4.21-4.24) |  |
|  |  |  |  |  |
| Alcohol intake |  |  |  |  |
| Non-drinker (141) | 4.23 (4.20-4.25) | 0.93 | 4.21 (4.18-4.25) | 0.39 |
| <15 g/d (435) | 4.23 (4.21-4.24) |  | 4.22 (4.21-4.24) |  |
| ≥15 g/d (85) | 4.22 (4.19-4.26) |  | 4.25 (4.19-4.31) |  |
|  |  |  |  |  |
| Smoking status |  |  |  |  |
| Never (283) | 4.23 (4.21-4.24) | 0.59 | 4.22 (4.20-4.25) | 0.98 |
| Past (294) | 4.23 (4.21-4.25) |  | 4.23 (4.21-4.25) |  |
| Current (81) | 4.21 (4.17-4.24) |  | 4.21 (4.17-4.26) |  |
|  |  |  |  |  |
| Family history of colorectal cancer |  |  |  |  |
| No (568) | 4.22 (4.21-4.24) | 0.56 | 4.22 (4.21-4.24) | 0.58 |
| Yes (93) | 4.23 (4.20-4.27) |  | 4.23 (4.20-4.27) |  |
|  |  |  |  |  |
| Age at blood draw |  |  |  |  |
| <60 yrs (330) | 4.23 (4.21-4.25) | 0.44 | 4.22 (4.20-4.25) | 0.94 |
| ≥60 yrs (331) | 4.22 (4.20-4.24) |  | 4.23 (4.20-4.25) |  |

Multivariate models are adjusted for age at blood draw, date of blood draw, race, height (continuous), fasting status, pack-years of smoking (continuous), body mass index (continuous), physical activity (in quartiles), family history of colorectal cancer (yes or no), history of colonoscopy or sigmoidoscopy (yes or no), alcohol intake (continuous), intake of red and processed meat (in quartiles), vitamin D intake (continuous), calcium intake (continuous), and aspirin use (non-users vs. ever users).
